# The m^6^A regulators in prostate cancer: molecular basis and clinical perspective

**DOI:** 10.3389/fphar.2024.1448872

**Published:** 2024-08-29

**Authors:** Yu Cao, Man Jia, Chunyan Duan, Zhihui Yang, Bo Cheng, Ronghao Wang

**Affiliations:** ^1^ Department of Biochemistry and Molecular Biology, School of Basic Medical Sciences, Southwest Medical University, Luzhou, China; ^2^ Department of Pathology, The Affiliated Hospital of Southwest Medical University, Luzhou, Sichuan, China; ^3^ Department of Urology, The Affiliated Hospital of Southwest Medical University, Luzhou, Sichuan, China

**Keywords:** PCa, N6-Methyladenosine, androgen deprivation therapy (ADT), androgen recepter, signaling pathway

## Abstract

Prostate cancer (PCa) is the second leading cause of cancer-related death among men in western countries. Evidence has indicated the significant role of the androgen receptor (AR) as the main driving factor in controlling the development of PCa, making androgen receptor inhibition (ARI) therapy a pivotal management approach. In addition, AR independent signaling pathways also contribute to PCa progression. One such signaling pathway that has garnered our attention is N6-Methyladenosine (m^6^A) signaling, which refers to a chemical modification on RNA with crucial roles in RNA metabolism and disease progression, including PCa. It is important to comprehensively summarize the role of each individual m^6^A regulator in PCa development and understand its interaction with AR signaling. This review aims to provide a thorough summary of the involvement of m^6^A regulators in PCa development, shedding light on their upstream and downstream signaling pathways. This summary sets the stage for a comprehensive review that would benefit the scientific community and clinical practice by enhancing our understanding of the biology of m^6^A regulators in the context of PCa.

## 1 Introduction

Prostate cancer (PCa), a malignancy originating from epithelial cells in the peripheral zone of prostate (Zhou et al., 2023), remains the second commonly diagnosed adenocarcinoma and the leading cause of cancer related deaths among men worldwide. The World Cancer Research Fund International survey estimated that 1,467,854 new cases of PCa were reported globally in 2022, resulting in approximately 397,430 deaths (Bray et al., 2024). Epidemiological studies have established that age (Godtman et al., 2022; Choi et al., 2018), race (Akaza et al., 2011; Jeong et al., 2016) and genetic factors (Bratt, 2002; Rebbeck, 2017; Thalgott et al., 2018) as the significant risk factors for PCa. PCa progresses through four stages, as determined by digital rectal examination (DRE) (Mottet et al., 2017), serum prostate specific antigen (PSA) level (Mottet et al., 2017) and pathological examination of biopsy samples (Kwon et al., 2020). Generally, low-grade and early localized PCa patients (PSA ≤10, Gleason score ≤6, or clinical stage T1-2a) are often managed by either radiotherapy or surgery. However, approximately 8% of PCa patients are viewed as advanced disease at their first diagnosis (Siegel et al., 2022). The cancer cells may spread from the prostate to other parts of the body, particularly the bones (Peng et al., 2017) and lymph nodes (Cai et al., 2011). In advanced stage, it may lead to urinary difficulty, hematuria, or pelvic pain. Targeting the androgen receptor (AR) signaling axis with androgen deprivation therapy (ADT) has been a primary treatment approach, showing favorable outcome (Dai et al., 2017; Davies et al., 2021; Guan et al., 2022; Jeon et al., 2023). Unfortunately, ADT is not curative and most patients will relapse within 2 years despite the low castrated level of serum testosterone. These patients are then considered to acquire castration-resistant PCa (CRPC), a highly lethal disease that accounts for the main mortality (Shigeta et al., 2019; Cai et al., 2023; Cheng et al., 2022; Wang et al., 2023a). Increasing evidence suggest that the reactivation of AR signaling plays a critical role in CRPC development, leading to the clinical approval of the second-generation AR antagonists such as enzalutamide (Enz) for managing this disease (de Bono et al., 2011; Agarwal et al., 2023; Powles et al., 2022; Wenzel et al., 2022). Despite the initial responses to this therapy, patients will eventually become Enz resistance owing to various mechanisms (Bennett et al., 2024; Liu et al., 2019; Zhang et al., 2020; Zheng et al., 2022). Additionally, approximately 30% of patients exhibit primary resistance to Enz treatment. These clinical findings collectively indicate limitations in the application of Enz.

Although AR is the main driving force for PCa progression, other signaling pathways, such as m^6^A signaling, are also involved in the regulation of PCa carcinogenesis and therapy resistance (He et al., 2022; Han et al., 2023). This review aims to comprehensively summarize the current understanding of the roles of RNA m^6^A regulators in PCa development and offer insights for further scientific research and clinical strategies.

## 2 Epitranscriptome and RNA m^6^A modification

**FIGURE 1:**
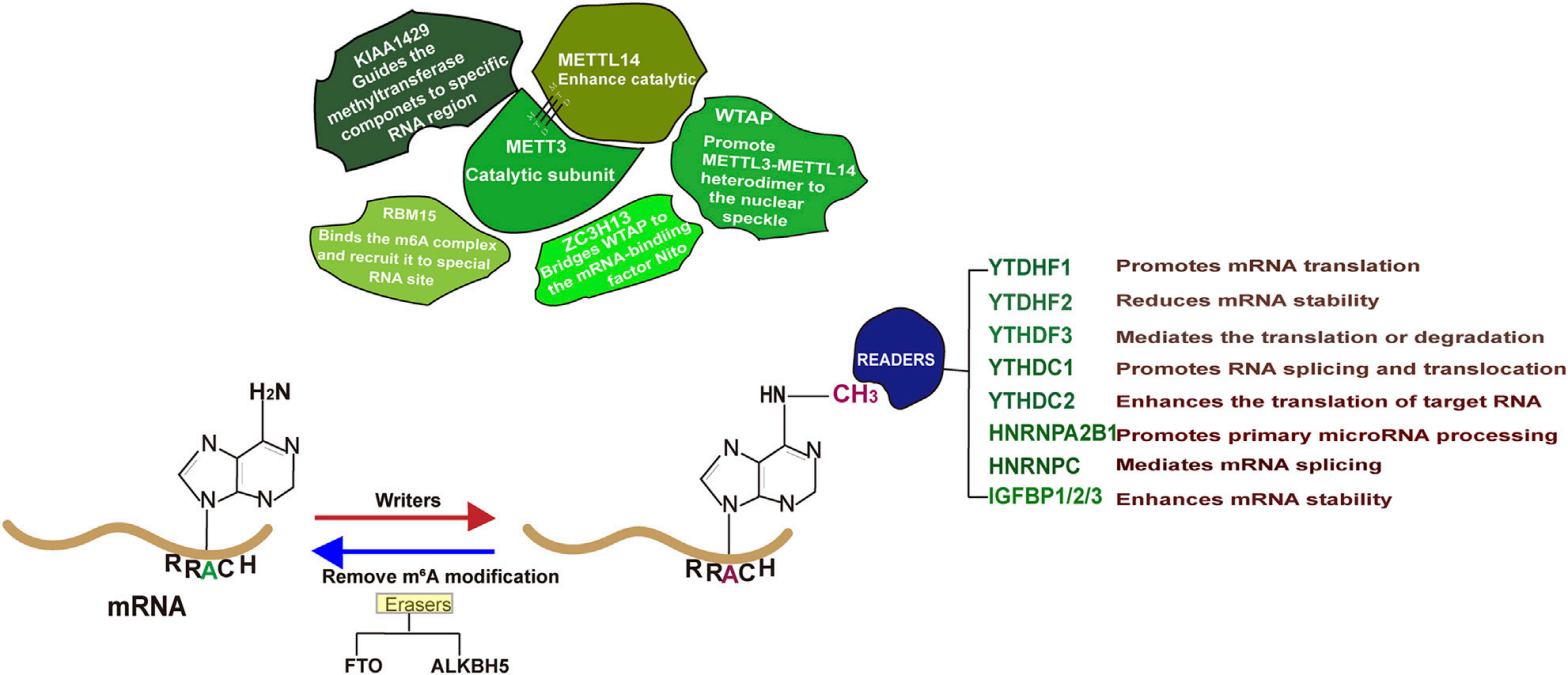
The general role of each individual m^6^A regulator in RNA metabolism.

Epitranscriptome, a biochemical modification on RNA, has received significant attention from scientists due to its critical roles in determining RNA metabolism as well as disease progression (Murakami and Jaffrey, 2022; Wang Y. et al., 2014; He et al., 2018; Bokar et al., 1997; Clancy et al., 2002; Sommer et al., 1978; Zhong et al., 2008). It is estimated that over 170 types of biochemical modifications occur in RNAs, with m^6^A as the major form (Wiener and Schwartz, 2021). Early identified in 1970s, m^6^A, the methyl-nitrogen at the position six of adenylate (Figure 1), has been reported to be functional (Wei et al., 1976; Desrosiers et al., 1974). The enzyme responsible for catalyzing RNA m^6^A modification, known as “Writer,” includes methyltransferase-like protein 3 (METTL3), METTL16, METTL5 and zinc finger CCHC type containing 4 (ZCCHC4) (Jiang et al., 2021). Among them, METTL3 methyltransferase complex, consisting of METTL3, METT14, WTAP (Wilms tumor 1 associated protein), Zinc finger CCCH-type containing 13 (ZC3H13), RNA-binding motif protein 15 (RBM15) and VIRMA (Vir Like M6A Methyltransferase Associated), is mainly responsible for the RNA m^6^A modification on the consensus sequence DRACH (D = A/G/U, R = A/G, H = A/C/U) (Linder et al., 2015; Zaccara et al., 2019; Huang et al., 2022; Raj et al., 2022; Wei et al., 2022; Ma et al., 2019). It is noting that the m^6^A modification is a reversible process and the methyl group can be removed by demetyltransferase (Eraser) such as obesity-associated protein (FTO) and Human AlkB homolog H5 (ALKBH5) (He et al., 2019). Once an RNA molecule is m^6^A modified, it becomes prone to recognition by a variety of proteins (Readers) and undergoes distinct fate (Zaccara et al., 2019). In general, m^6^A modification on mRNA enables to influence its splicing, stability or translation. Recent advances in this area suggest that m^6^A regulators play vital roles in various human cancers, including PCa (Zhu W. et al., 2023). Studies have demonstrated that m^6^A level in PCa is disease stage dependent and m^6^A regulators are causally related to PCa growth, metastasis and targeted therapy resistance (Lothion-Roy et al., 2022). Therefore, there is a need to comprehensively summarize the molecular basis of m^6^A regulator mediated PCa carcinogenesis, which will definitely provide valuable insights for future scientific investigations and clinical applications.

### 2.1 METTL3/METTL14 m^6^A writer in PCa

METTL3/METTL14 methyltransferase complex is primarily responsible for the m^6^A modification of RNAs (Wang P. et al., 2016; Wang X. et al., 2016; Śledź and Jinek, 2016; Choe et al., 2018; Geula et al., 2015; Lin et al., 2016). Several studies have demonstrated that the expression levels of METTL3 and METTL14 are elevated in PCa as compared to normal tissues, acting as tumor promoting driver (Xu and Ge, 2022). Additionally, castration resistance perpetuates the increased expression levels of these two proteins (Wu et al., 2021). Supported by *in vitro and in vivo* evidence, METT3 complex promotes PCa growth and metastasis via catalyzing m^6^A modification of various mRNAs and non-coding RNAs (ncRNAs).

#### 2.1.1 The targets and biological functions of METTL3 in PCa

Advance in this field has led to the identification of a wide range of m^6^A targets. To date, mRNAs including c-Myc (Liu et al., 2022), USP4 (Chen et al., 2021a), LEF1 (Ma et al., 2020), DDIT4 (Zhao Y. et al., 2024), PRSS8 (Zhao X. et al., 2024), ZFHX3 (Hu et al., 2024) and others have been viewed as m^6^A targets in PCa (Table 1 **)**. In addition, ncRNAs, a class of RNAs without protein coding potential but proven to be physiologically and pathologically functional in a variety of disease models, are also potential targets of METTL3 complex in PCa. Specifically, lncRNAs (NEAT1 (Wen et al., 2020), MALAT1 (Mao et al., 2022), SNHG7 (Liu et al., 2022), PVT1 (Chen B. et al., 2023)), miRNAs (miR-139-5p (Azhati et al., 2023), pre-miR-25 (Qi et al., 2023), pre-miR-93 (Qi et al., 2023), miR-148-3p (Li G. et al., 2023)) and circRNAs (circDDIT4 (Kong et al., 2023), circABCC4 (Huang C. et al., 2023), circRBM33 (Zhong et al., 2023) and hsa_circ_0003258 (Yu et al., 2022)) have been reported as the substrates of METTL3. The m^6^A modification site, the RNA fate, the specific reader and the biological consequence of each individual RNA molecule are summarized and listed in Table 1. The literature illustrate a high expression level of METTL3 in PCa, implying it may contribute to PCa development. Indeed, by catalyzing m^6^A modifications of RNAs, METTL3 promotes PCa survival, metastasis and therapy resistance. For example, ubiquitin-specific protease 4 (USP4) was identified by Chen et al. as one target of METTL3 by the m^6^A-RIP (RNA immunoprecipitation) qPCR. Upon being m^6^A modified at the A2696, USP4 mRNA is recognized by YTH N (6)-Methyladenosine RNA Binding Protein 2 (YTHDF2) and undergoes degradation, subsequently leading to the protein degradation of ELAV like RNA-binding protein 1 (ELAV1). As a consequence, METTL3 mediated ELAV1 degradation increases ARHGDIA expression and promotes PCa growth and metastasis. Thus, targeting METTL3 by shRNAs powerfully attenuated PCa development *in vitro and in vivo* (Chen et al., 2021a).

**TABLE 1:** The m^6^A targets in PCa.

Regulators	Target	m^6^A site	Reader	Biological consequence
METTLE3	c-Myc	NA	NA	Increase c-Myc mRNA transcription
	USP4	3′-UTR	YTHDF2	Increase USP4 mRNA degradation
	LEF1	NA	IGF2BP2	Increase LEF1 protein level
	Gli	NA	NA	Increase Gli protein level
	KIF3C	NA	IGF2BP1	Increase KIF3C mRNA stability
	ITGB1	NA	NA	Increase ITGB1 mRNA stability
	CTNNB1	3′-UTR	NA	Decrease CTNNB1 mRNA stability
	NAP1L2	NA	HNRNPC	Increase NAP1L2 mRNA stability
	HRAS	3′-UTR	IGF2BP2	Increase mRNA stability
	MEK2	5′-UTR	IGF2BP2	Promote protein transcription
	AR	3′-UTR	YTHDF3	Regulate AR spling
	CLIC4	3′-UTR	NA	Increase CLIC4 mRNA stability
	ERG2	NA	NA	Increase ERG2 mRNA stability
	PLK1	3′-URT	YTHDF1	Increase PLK1 mRNA transcripotion
	LHPP	NA	YTHDF2	Increase LHPP mRNA degradation
	NKX3-1	NA	YTHDF2	Increase NKX3-1 mRNA degradation
	PRMT6	NA	NA	Stability
	SIAH1	NA	NA	Increase SIAH1 mRNA degradation
	ARHGDIA	NA	NA	Increase ARHGDIA mRNA stability by regulating ELAVL1 expression
	PCAT6	NA	IGF2BP2	Increase PCAT6 mRNA stability
	lncRNA SNHG7	NA	NA	Increase SNHG7 RNA stability
	lncRNA NEAT1	5′-UTR 3′UTR	NA	Increase NEAT1 RNA stability
	lncR MALAT1	NA	NA	Increase MALATI RNA stability
	lncR PVT1	NA	NA	Increase PVT1 RNA stability
	miR-139-5p	NA	NA	Increase miR-139-5p RNA stability
	pre-miR-25	NA	HNRNPA2B1	Promote pre-miR-25 maturation
	pre-miR-93	NA	HNRNPA2B1	Promote pre-miR-93 maturation
	miR-148-3p	NA	NA	Promote pre-miR-148-3p maturation
	circDDIT4	3′-UTR 5′-UTR	NA	Promote circDDIT4 circularization
	circABCC4	NA	IG2BP2	Increae circABCC4 RNA stability
	circRBM33	NA	NA	NA
METTL14	THBS1	NA	YTHDF2	Increase THBS1 mRNA degradatiom
FTO	CLIC4	3′-UTR	NA	Increase CLIC4 mRNA stability
	MC4R	3′-UTR	NA	Increase MC4R mRNA degradation
	ERG2	NA	NA	Increase ERG2 mRNA stability
	miR-139-5p	NA	NA	Increase miR-139-5p stability
	DDIT4	3′-UTR	IGFBP2/3	Increase DDIT4 mRNA stability
	ZFHX3	NA	—	Increase the stability of ZFHX3 transcripts
ALKBH5	SIAH1	NA	NA	Increase SIAH1 mRNA degradation
	PRMT6	NA	NA	Suppress PRMT6 level
IGF2BP1/2/3	LEF1	NA	—	Increase LEF1 protein level
	LDHA	3′-UTR	—	Increase LDHA mRNA stability
	IGF1R	NA	—	Icrease IGF1R mRNA stability via PCAT6/IGF2BP2 complex
	HMGCS1	NA	—	Increase HMGCS1 mRNA stability
	HDAC4	NA	—	Increase HDAC4 mRNA stability
YTHDF1	PLK1	3′-UTR	—	Increase PLK1 mRNA transcripotion
	TRIM44	NA	—	Increase TRIM44 level
YTHDF2	USP4	3′-UTR	—	Increase USP4 mRNA degradation
	MOB3B	NA	—	Increase MOB3B mRNA degradation
	LHPP	NA	—	Increase LHPP mRNA degradation
	NKX3-1	NA	—	Increase NKX3-1 mRNA degradation
	PRSS8	NA	—	Increase PSRR8 mRNA degradation
YTHDC1	CD44	NA	—	Increase CD44 splicing
	HOBX13	NA	—	Increase HOBX13 mRNA stability
HNRNPA2B1	miR-93-5p	NA	—	Promote pre-miR-93 maturation
	miR-25-3p	NA	—	Promote pre-miR-25 maturation

METTL3 has also been implicated in the regulation of glycolysis in PCa by adding methyl groups to lncRNA SNHG7, thereby enhancing its stability. Consequently, SNHG7 interacts with SRSF1 to promote the expression of c-Myc, a transcription factor related to glycolysis by regulating the expression of various genes (Liu et al., 2022). Furthermore, Li et al. observed an increased level of METTL3 in enzalutamide resistant PCa cells, implying it may be a causal factor determining enzalutamide resistance. Indeed, METTL3 could activate MAPK signaling via catalyzing the m^6^A modifications of HRAS and MEK2 mRNAs to bypass AR inhibition therapy (Li Y. et al., 2023). Based on this, we can envision a potential combined therapy involving enzalutamide and a specific METTL3 inhibitor for the treatment of CRPC patients.

To summarize, METTL3 plays a tumor promoting role in PCa progression and targeted therapy resistance at least by catalyzing some oncogenes (c-Myc) (Liu et al., 2022) and core component of multiple signaling pathways including WNT signaling (CTNNB1) (Zhang S. et al., 2023), Hedgehog signaling (Gli) (Cai et al., 2019), MAPK signaling (HRAS, MEK2) (Li Y. et al., 2023). Whether METTL3 has an impact on other signaling pathways that influence PCa remains to be further explored through the continuous identification of its targets.

#### 2.1.2 The role of METTL14 in PCa

As a critical component of METTL3 complex (Liu et al., 2014), METTL14 is also clinically correlated to PCa prognosis. Functionally, METT14 increases PCa proliferation *in vitro and in vivo*, largely through its regulation of thrombospondin 1 (THBS1) mRNA based on the analysis of RNA-seq and MeRIP (Methylated RNA Immunoprecipitation)-seq. Mechanistically, the m^6^A mark of THBS1 mRNA in the presence of METTL14 is recognized by YTHDF2, predisposing THBS1 mRNA to degrade (Wang Y. et al., 2022). However, in our opinion, the observed phenotype caused by METTL14 knockdown may be METTL3 complex dependent since the main role of METTL14 is to enhance METTL3 activity. It is anticipated that METTL14 deficiency severely impairs the enzymatic activity of METTL3 complex, leading to abnormal m^6^A modifications and impeding PCa growth. Nevertheless, it is plausible that METTL14 may have a METTL3 complex independent role in PCa, and this hypothesis can be tested by proposing experiments in METTL13-KO cells.

#### 2.1.3 Other m^6^A writers in PCa

METTL16, another methyltransferase responsible for the m^6^A modifications of snRNAs and some lncRNAs (Pendleton et al., 2017; Shima et al., 2017; Warda et al., 2017), has not been investigated in PCa yet. It is noteworthy that the splicing events in PCa, especially CRPC, are highly active, leading to the generation of splicing products such as androgen receptor variant 7 (ARv7). Given the facts that 1) METTL16 is a m^6^A writer of *MALAT1* (Ruszkowska et al., 2018); 2) *MALAT1* mediated ARv7 signaling contribute to enzalutamide resistance (Wang et al., 2017), it would be interesting enough to explore the potential connections of METTL16 with anti-androgen resistance. Besides, whether METTL5 and ZCCHC4, the enzymes adding methyl group to ribosome RNAs (rRNAs), play contributing roles in PCa development is worthy of future investigations (van Tran et al., 2019).

### 2.2 M^6^A eraser

As mentioned above, it is important to note that m^6^A modification is a reversible process. FTO and ALKBH5 are the two well-known demethylases responsible for the removal of m^6^A in RNA molecule.

#### 2.2.1 FTO in PCa

**FIGURE 2:**
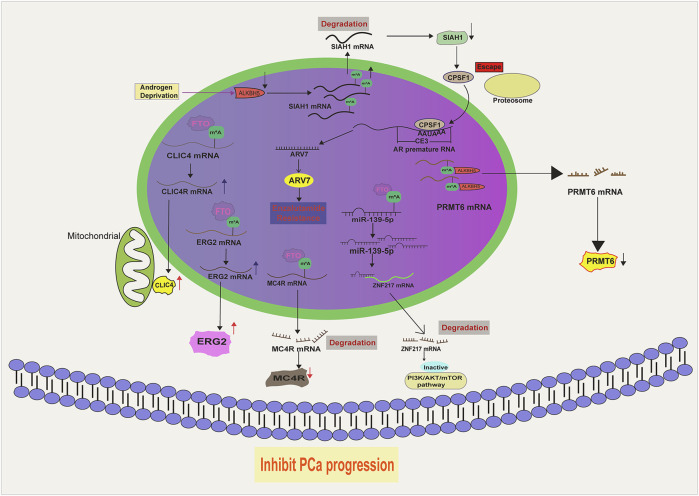
The molecular basis of FTO and ALKB5 in PCa.

FTO was initially viewed as a demethylase of methylated DNAs (Gerken et al., 2007). However, subsequent studies have unraveled its preference for selecting RNAs, especially snRNAs (small nuclear RNAs), as substrates. Specifically, FTO recognizes m^6^A_m_ (N6,2′-O-dimethyladenosine) in snRNAs and removes the methyl base (Wei et al., 2018; Mauer et al., 2017; Mauer et al., 2019) (Figure 2). Nevertheless, upcoming evidence suggests that FTO also holds a weak activity towards m^6^A, indicating its abnormal expression may impair the mRNAs metabolism (Li Y. et al., 2022).

FTO is expressed at a lower level in PCa as compared to normal prostate tissues (Zhu et al., 2021). Moreover, PCa patients with low FTO expression often experience advanced disease and poor survival, suggesting that it acts as a tumor suppressor during PCa development (Wang Z. et al., 2022). Indeed, FTO depletion remarkably facilitates PCa malignancy *in vitro and in vivo* by increasing the total m^6^A level. Mechanistically, the loss of FTO increases the m^6^A levels of chloride intracellular channel 4(CLIC4) and ERG2, which are two tumor suppressors in PCa, accelerating their degradation (Zou et al., 2022). Moreover, melanocortin 4 receptor (MC4R), identified as another substrate of FTO in PCa, exhibits a high expression level owing to its abundant m^6^A mark resulting from FTO loss (Li and Cao, 2022). A recent literature has also demonstrated that FTO enables to decrease Zinc Finger Protein (ZNF217) expression by stabilizing miR-139-5p level via an m^6^A dependent manner. Consequently, FTO mediated ZNF217 reduction inactivates PI3K/AKT/mTOR signaling, impeding PCa progression. Collectively, these results suggest that FTO exerts a tumor-suppressing role in PCa progression via altering the m^6^A level of a specific RNA population (Figure 2). Intriguingly, the biological function of FTO is cancer context dependent. For instance, in renal cell carcinoma (Zhang et al., 2022), bladder cancer (Tao et al., 2021), breast cancer (Xu et al., 2020) and leukemia (Li et al., 2017), FTO functions as a tumor promoting factor. We postulate that the targets of FTO in different cancer models vary and determine the its functional identity. Therefore, it will be necessary to devote more efforts to identify the substrates of FTO in order to fully understand its biology in PCa.

#### 2.2.2 ALKBH5 in PCa

ALKBH5, a member of the ALKB Family, specifically catalyzes the removal of the m^6^A modification on small nuclear RNAs (Figure 2). In contrast to FTO, ALKBH5 does not exhibit activity towards m^6^A_m_ (Mauer et al., 2017; Mauer et al., 2019; Koh et al., 2019). Despite appearing to be an oncogene in cancer development due to its reported induction by hypoxia (Dong et al., 2021; Thalhammer et al., 2011), ALKBH5 actually functions to attenuate PCa growth. A study by Li et al. revealed that ALKBH5 has a marginal expression in PCa tissues and its overexpression apparently suppresses PCa cell growth and cell invasion via reducing the expression level of protein arginine methyltransferase 6 (PRMT6) via an m^6^A dependent manner (Li X. et al., 2023) (Figure 2). Similarly, Xia et al. (2022) observed a reduction of ALKBH5 in PCa cells with androgen deprivation. Consequently, SIAH1 mRNA is degraded due to the elevated m^6^A level resulted from the reduction of ALKBH5. Being a target of SIAH1, cleavage and polyadenylation specificity factor 1 (CPSF1) evades the proteosomal degradation and binds to the enriched AAUAAA sequence in the CE3 (cryptic exon 3) region of the AR premature mRNA, thereby facilitating its splicing to ARv7, a potent AR variant playing a critical role in castration resistance (Figure 2). These evidence suggest that ALKBH5 is a tumor suppressor in PCa. Again, the identification of ALKBH5 targets should be pursued if we want to fully understand its PCa associated biology. Potentially, an ALKBH5 agonist, if available in the future, may offer clinical benefits for PCa patients.

### 2.3 M^6^A readers

#### 2.3.1 YTHDF family proteins

The YTHDF family consists of YTHDF1, YTHDF2 and YTHDF3 (Patil et al., 2018). Although sharing similar identity at the amino acid sequence, they have distinct biological effects on their targets (Patil et al., 2018; Chen L. et al., 2023). An early study has demonstrated that YTHDF1 binds to the m^6^A modified 3′-UTR of mRNAs, enhancing their translation (Wang et al., 2015; Wang X. et al., 2014). In contrast, YTHDF2 binds to its targets, leading to their instability and degradation (Li et al., 2020). While YTHDF3 has the capacity to influence both translation and stability of its bound targets (Shi et al., 2017).

**FIGURE 3:**
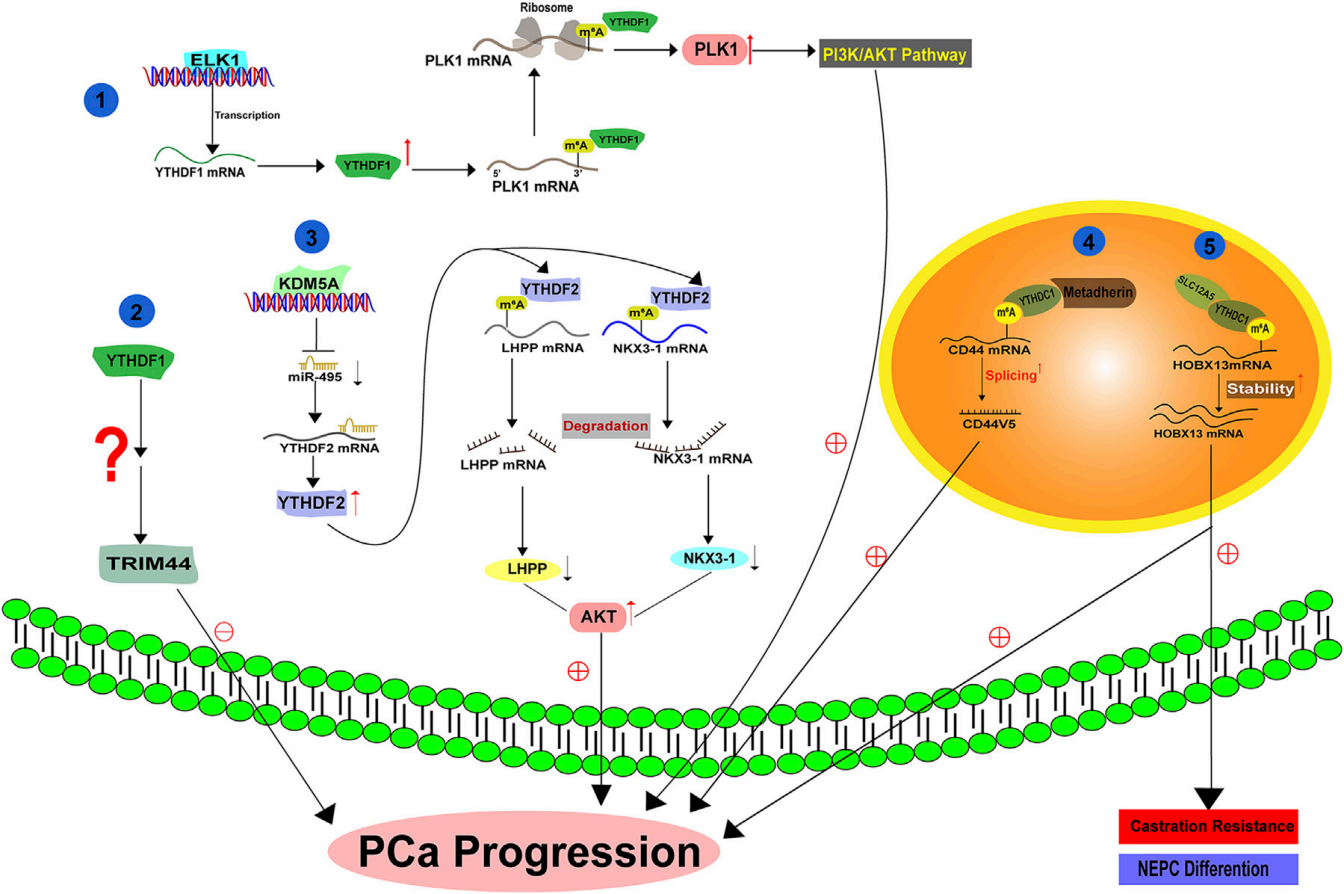
The molecular basis of YTH family protein in PCa.

Li et al. (2021) demonstrated that YTHDF1 exhibits high expressionin in PCa and its level is correlated with disease prognosis. Knockdown of YTHDF1 significantly represses PCa survival, migration and invasion by regulating tripartite motif containing 44 (TRIM44) (Figure 3). Agreeably, another literature also suggested that YTHDF1, which is transcriptionally controlled by ELK1, facilitates PCa development *in vitro* and *in vivo* by activating polo-like kinase1 (PLK1) mediated PI3K-AKT signaling. Mechanistically, YTHDF1 binds to the m^6^A modified 3′-UTR of PLK1 mRNA and enhances its translation (Li P. et al., 2022) (Figure 3). YTHDF2 is also increased in PCa and its high expression indicates a poor overall survival. YTHDF2 exerts its oncogenic effect at least by mediating the instability and degradation of Phospholysine Phosphohistidine Inorganic Pyrophosphate Phosphatase (LHPP) and Homeobox Protein NK-3 Homolog A (NKX3–1) mRNAs, leading to the activation of AKT signaling and PCa progression (Li et al., 2020) (Figure 3). Therefore, upregulation of YTHDF2 via Lysine Demethylase 5A (KDM5A) mediated miR-495 reduction could drive PCa progression *in vitro and in vivo* (Du et al., 2020) (Figure 3). As another YTHDF family protein, YTHDF3 has not been functionally characterized in PCa. A recent literature has illustrated that YTHDF3 can bind the m^6^A modified AR mRNA and increase its translation in PCa cells (Somasekharan et al., 2022) (Figure 3). Given the significance of AR in PCa, it is tempting to hypothesize that YTHDF3 may act as an oncogenic protein to facilitate PCa growth, although this hypothesis requires experimental supports.

#### 2.3.2 YTHDC1 and YTHDC2 in PCa

Primarily localized in the nucleus (Hartmann et al., 1999), YTHDC1 has been reported to regulate the splicing and nuclear export of the targets with m^6^A modification (Widagdo et al., 2022; Roundtree et al., 2017). The splicing activity of YTHDC1 is attributed to its association with serine and arginine-rich splicing factor 3 (SRSF3), an important splicing factor that regulates exon inclusion (Xiao et al., 2016). A recent study by Cheng et al. (2021) reported that YTHDC1 undergoes phase separation to control gene expression via various means, suggesting its diverse biological functions. In PCa, YTHDC1 interacts with the oncogene protein MTDH (Metadherin), facilitating the generation of splicing product CD44v5 and promoting PCa malignancy (Luxton et al., 2019) (Figure 3). In addition, YTHDC1 can also complex with SLC12A5 (a neuron-specific potassium-chloride co-transporter) and enhance its oncogenic function. As a result, YTHDC1-SLC12A5 complex promotes PCa progression, castration resistance and neuroendocrine differentiation by recognizing and stabilizing m^6^A modified Homeobox B13 (HOXB13) mRNA (Yuan et al., 2023) (Figure 3). Considering the highly active splicing process during the progression of PCa to an advanced stage, we surmise that YTHDC1 may hold a fundamental role in the development of PCa by regulating the amount of various splicing products in an m^6^A dependent manner.

Although YTHDC2 is not ubiquitously expressed and its high abundance is observed in testes (Bailey et al., 2017; Hsu et al., 2017; Jain et al., 2018), it does not exclude the possible causal involvement of YTHDC2 into the development of other diseases. Notably, a high expression of YTHDC2 is observed in PCa as compared to BPH (Benign prostatic hyperplasia) and normal prostate tissues. Experimental results have shown that YTHDC2 induction substantially promotes PCa cell growth and invasion (Song et al., 2023). Nevertheless, the underlying mechanism by which YTHDC2 drives PCa progression has not been investigated, and the exploration of the downstream targets of YTHDC2 in PCa remains an open area. Since the early claim suggested that YTHDC2 exhibits a very weak affinity towards m^6^A motif (Wojtas et al., 2017), it is reasonable to speculate that YTHDC2 may have non-m^6^A targets.

#### 2.3.3 IGF2BP family proteins

**FIGURE 4:**
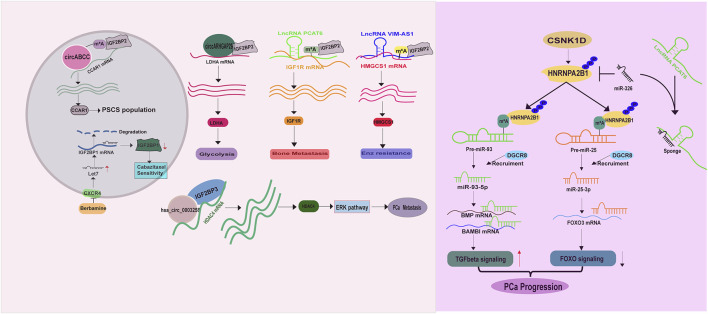
The molecular basis of IGF2BP and HnRNP family proteins in PCa.

IGF2BP proteins enable to recognize m^6^A targets or non m^6^A targets and to increase their stabilities (Jiang et al., 2021; Huang et al., 2018; Lan et al., 2021), thus having a great impact on PCa development. A literature has demonstrated an increase of IGF2BP1 expression in prostate cancer stem cells (PCSCs), contributing to cabazitaxel resistance. Thus targeting CXCR4 (C-X-C chemokine receptor type 4)/let-7 mediated IGF2BP1 induction in PCSCs by Berbamine could restore PCa response to cabazitaxel treatment (Wang et al., 2024). Similarly, IGF2BP2 has been reported to recognize m^6^A labeled circABCC (circular ATP Binding Cassette Subfamily C Member), a prerequisite for stabilizing Cell Division Cycle And Apoptosis Regulator 1 (CCAR1) mRNA, expanding PCSCs population (Huang C. et al., 2023) (Figure 4). Besides, IGF2BP2 exerts a tumor promoting role in altering PCa metabolism, bone metastasis and targeted therapy resistance. A study from Jiang et al. unraveled that IGF2BP2 increases its binding affinity to Lactate dehydrogenase A (LDHA) mRNA in the presence of circARHGAP29 (circular Rho GTPase Activating Protein 29), thereby enhancing glycolytic metabolism (Jiang et al., 2022) (Figure 4). Another study demonstrated that IGF2BP2 is recruited by m^6^A modified lncRNA PCAT6 (Prostate Cancer Associated Transcript 6) to interact with IGF1R (Insulin-Like Growth Factor I Receptor) mRNA, resulting in its stabilization and the promotion of PCa bone metastasis (Lang et al., 2021) (Figure 4). Moreover, IGF2BP2 can confer enzalutamide resistance via binding to and stabilizing 3-Hydroxy-3-Methylglutaryl-CoA Synthase 1 (HMGCS1) mRNA in the presence of lncRNA VIM-AS1 (VIM Antisense RNA 1) (Shi et al., 2023) (Figure 4). IGF2BP3 also serves as an oncogene in PCa, as a study illustrated its ability to combine with hsa_circ_0003258 to directly enhance the stability of histone deacetylase 4 (HDAC4) mRNA, consequently activating ERK signaling pathway to drive PCa metastasis (Yu et al., 2022) (Figure 4).

Together, these evidences suggest that IGF2BP proteins support PCa survival and hasten its malignancy via stabilizing a wide range of mRNAs. Furthermore, it is evident that the impact of IGF2BP proteins on mRNA stabilization is m^6^A and non-m^6^A dependent, suggesting that classifying and identifying the targets of IGF2BP proteins based on the m^6^A status may aid in comprehending their biologies in PCa.

#### 2.3.4 HnRNP family proteins

Accumulating evidence have demonstrated that the heterogeneous nuclear ribonucleoproteins (HnRNP) such as HnRNPC, HnRNPG and HNRNPA2B1 are direct or indirect readers of m^6^A labeled RNAs, especially miRNAs (Wang et al., 2020; Liu et al., 2015; Spitale et al., 2015; Liu et al., 2017; Wu et al., 2018). In PCa, elevated HnRNPC expression is closely correlated with tumor stage, tumor grade and the overall survival (Wang et al., 2021). Functionally, HnRNPC promotes PCa proliferation and metastasis (Cheng et al., 2023). Moreover, a high level of HNRNPA2B1 is also examined in PCa. HNRNPA2B1 binds to the m^6^A marks in several miRNA precursors (miR-93-5p (Qi et al., 2023; Sun et al., 2023), miR-25-3p (Qi et al., 2023)) and facilitates their processing and maturation via recruiting DGCR8 (DiGeorge syndrome critical region gene 8) (Sun et al., 2023), driving PCa development (Figure 4). For this point of view, molecules enabling to regulate HNRNPA2B1 expression is supposed to have a considerable impact on PCa survival and metastasis. As expected, casein kinase 1 delta (CSNK1D) phosphorylates and stabilizes HNRNPA2B1 protein, exacerbating PCa malignancy (Qi et al., 2023) (Figure 4). The lncRNA PCAT6 also has capacity to increase HNRNPA2B1 expression via acting as sponge of miR-326 to facilitate PCa neuroendocrine differentiation (Liu B. et al., 2021) (Figure 4). However, the role of another m^6^A reader, HnRNPG, has not been investigated in PCa.

## 3 The upstream signaling pathways regulating m^6^A regulators

**FIGURE 5:**
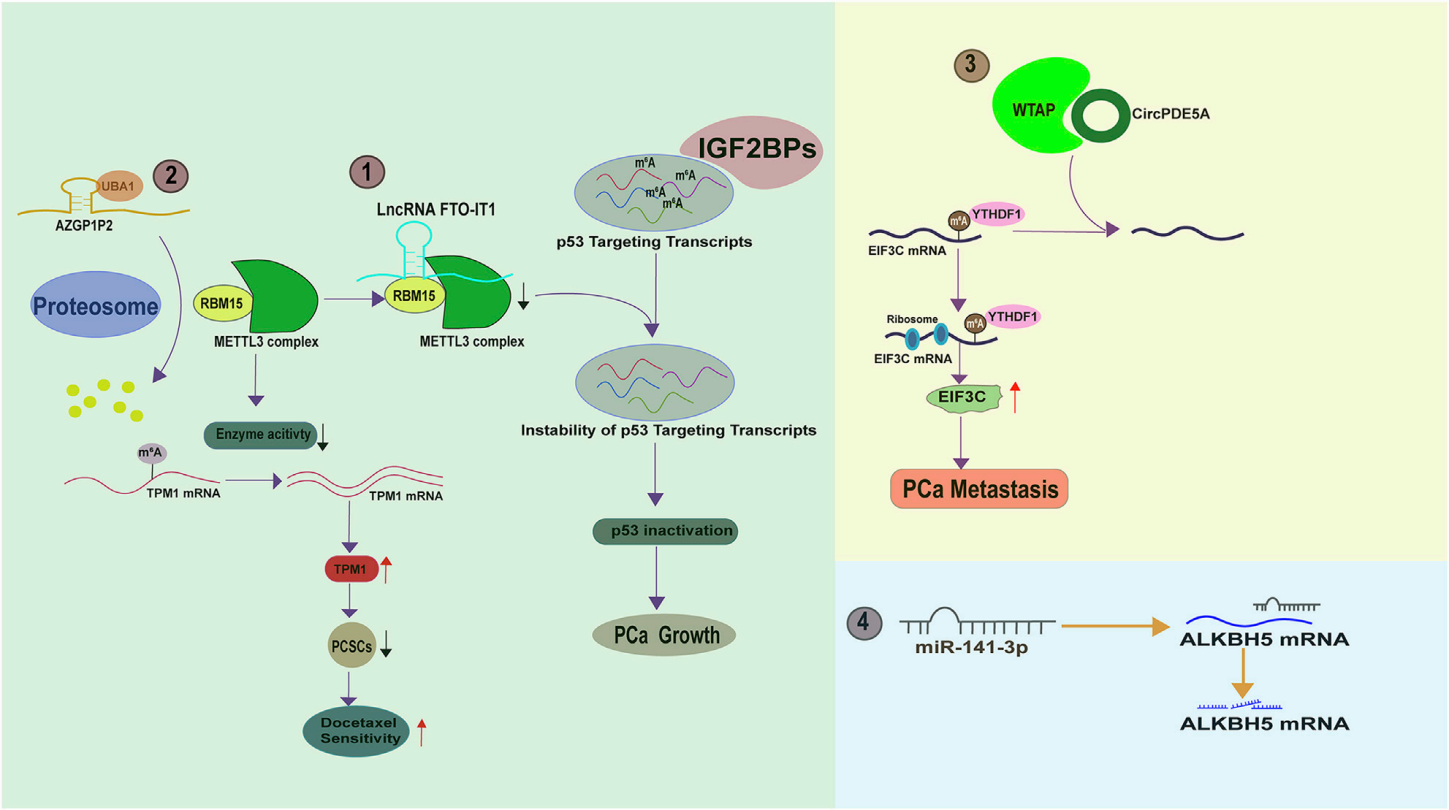
The upstream signaling molecules regulating m^6^A regulator in PCa.

A literature suggest that the total m^6^A levels are gradually increased as PCa progresses from the localized mass to the metastatic disease (Wan et al., 2022), indicating the existence of a molecular network upstream of m^6^A regulators in PCa. Understanding this network may provide insight into novel strategies to improve the efficacy of current therapies. A fascinating study by Zhang et al. demonstrated that FTO-IT1 (FTO intronic transcript 1), a lncRNA transcribed from the intron 8 of *FTO* gene focus, downregulates the transcript levels of several p53 targeting genes such as FAS (Fas Cell Surface Death Receptor), TP53INP1 (Tumor protein p53-inducible nuclear protein 1), SESN2 (Sestrin2), and MDM2 (Mouse double minute 2 homolog), thereby recapitulating p53 inactivation. Results from RNA pull down and subsequent mass spectrum analysis illustrated that FTO-IT1 directly interacts with RBM15 but not other m^6^A regulator to inhibit the methyltransferase activity of METTL3 complex. As a sequence specific RNA binding protein, RBM15 fails to bind p53 targeting transcripts for m^6^A modification in the presence of FTO-IT1, leading to their failure to be recognized by IGF2BP proteins. Thus, FTO-IT1 knock-out specifically boosts the m^6^A levels of p53 targeting transcripts by releasing RBM15 mediated m^6^A “writer” activity and caused PCa cell growth arrest (Zhang J. et al., 2023) (Figure 5). Another study by Wang et al. also documented that RBM15 can be regulated by AZGP1P2, a pseudogene of AZGP2. According to the data, AZGP1P2 binds and recruits UBA1 (Ubiquitin Like Modifier Activating Enzyme 1) as a E1 conjugating enzyme for RBM15 degradation. As a result, the m^6^A of RBM15 recognized TPM1 mRNA (tropomyosin 1) at its coding region is erased and TPM1 mRNA is stabilized. TPM1 induction by AZGP1P2 functions as a tumor suppressor to sensitize PCa cells to docetaxel therapy via eradicating the population of prostate cancer stem cells (PCSCs) (Wang et al., 2023b) (Figure 5).

WTAP, a known m^6^A regulator, is reportedly regulated by circPDE5A, a circular form of exon 2 and exon 3 of PDE5A (Phosphodiesterase 5A). CircPDE5A binds WTAP and disrupts its mediated m^6^A modification of eukaryotic translation initiation factor 3c (EIF3C) (Figure 5). Therefore, circPDE5A inactivation in CRPC leads to an m^6^A increase of EIF3C mRNA, which is subsequently recognized by YTHDF1 and has an enhanced translation efficiency, eventually promoting PCa metastasis (Ding et al., 2022). Moreover, it has been documented that the m^6^A “eraser” ALKBH5 is a direct target of miR-141-3p (Li X. et al., 2023) (Figure 5). In the future, we can anticipate the identification of more upstream molecules that affect m^6^A regulators. Armed with this knowledge, we can effectively silence m^6^A signaling by targeting these upstream molecules.

## 4 The cross-talk between RNA m^6^A modification and AR signaling

Androgen receptor (AR), a member of steroid hormone receptors, has been acknowledged as the key driving factor determining PCa development for decades (Tang et al., 2021). Structurally consisting of N-terminal, DNA binding domain, Hinge region and Ligand binding domain, AR responds to dihydrotestosterone (DHT) and translocates into nucleus as dimer to regulate the transcription of numerous genes (Tan et al., 2015). Owing to the significant role of AR in PCa development, for a long time, AR signaling inhibition has been the main strategy for PCa management.

**FIGURE 6:**
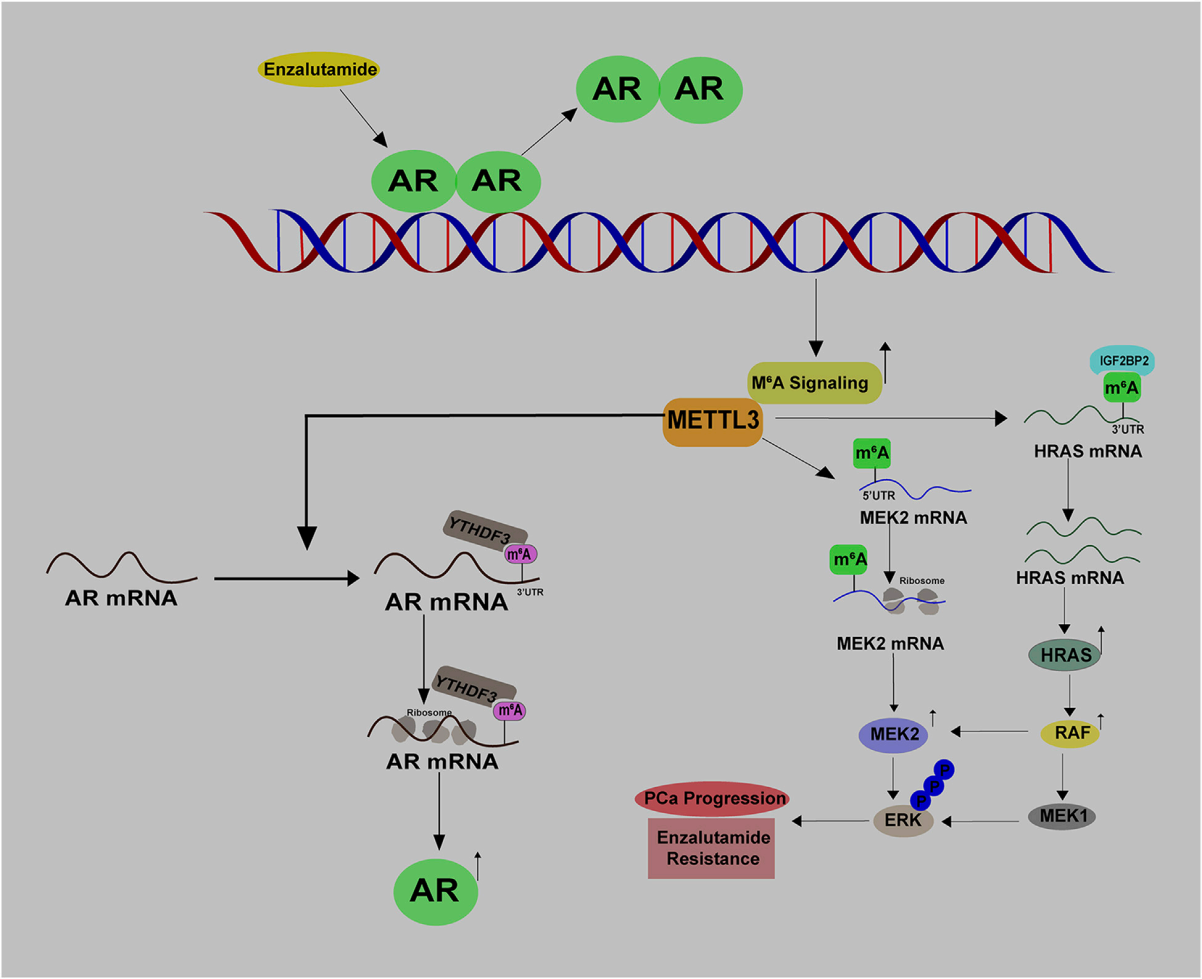
The crosstalk between m^6^A signaling and AR signaling in PCa.

Androgen deprivation therapy (ADT) has been utilized as the golden mean to treat PCa for many decades, with promising clinical outcomes. Li et al. have uncovered a direct link between RNA m^6^A modification and androgen receptor (AR) signaling. Their research showed that ADT with enzalutamide treatment leads to an increase in METTL3 expression and the total m^6^A levels, suggesting METTL3 mediated m^6^A modification may contribute to the acquired Enz resistance (Figure 6). By performing MeRIP-seq and RNA-seq, the authors identified that METTL3 directly mediates m^6^A modifications of HRAS and MEK2 mRNAs. Mechanistically, HRAS mRNA with m^6^A at its 3′-UTR is much more stable, and MEK2 mRNA with m^6^A at 5′-UTR has a higher translation potential as compared to the corresponding non-modified controls. As a result, MAPK signaling is activated and bypasses AR signaling inhibition to promote PCa growth (Li Y. et al., 2023) (Figure 6). Therefore, activation of m^6^A signaling serves as a self-protective mechanism in response to AR inhibition, providing a non-AR survival source for PCa growth. Given the fact that enzalutamide is an anti-androgen drug specifically preventing the transcription activity of AR, it will be intriguing to explore whether AR enables to control the expression of m^6^A regulators at the chromatin level, thus affecting the m^6^A signaling.

Reciprocally, m^6^A signaling also has a great impact on AR signaling. Evidence from Haigh et al. suggested that METTL3 inhibition by siRNAs could substantially impair androgen regulated transcriptome in PCa (Haigh et al., 2022). Additionally, in early 2022, Somasekharan et al. (2022) discovered that AR mRNA is a direct target of METTL3 and its translation is potentiated with the m^6^A modification at 8953A (Figure 6). By connecting these two findings, we speculate that METTL3 may affect androgen regulated transcriptome via directly methylating AR mRNA. Given that CRPC expresses more abundant AR protein than primary PCa, it is hypothesized that METTL3-mediated AR mRNA translation at least partially accounts for this phenomenon (Wu et al., 2021). Therefore, targeting METTL3 may alleviate the reactivation of AR signaling and aid in overcoming CRPC progression. In summary, there exists a reciprocal regulation between AR signaling and m^6^A signaling in PCa.

## 5 Clinical implications of RNA m^6^A modification in PCa and future perspectives

The clinical significance of RNA m^6^A modification should be acknowledged owing to its close relationship with PCa initiation, progression and therapy resistance. METTL3 and METTL14, the main components of m^6^A “writer,” elevate their expression when prostate epithelial cells become malignant (Xu and Ge, 2022) (Table 2). A continual rise in METTL3 and METTL14 expression is observed in CRPC disease (Wu et al., 2021). Conversely, the expression levels of m^6^A “eraser,” FTO and ALKBH5, display an opposite trend (Fang et al., 2022). In line with this, Lu et al. found that m^6^A modification levels are elevated in metastatic PCa as compared to the primary control, as evidenced by MeRIP-seq and RNA-seq on 4 metastatic PCa, 4 primary PCa tumors and 4 benign prostate hyperplasia (BPH). Importantly, they also reported that PCa patients with high m^6^A-modified mRNA (MMM) score experience shorter biochemical recurrence free survival and have a poor response to androgen signaling inhibition therapy as compared to the patients with a low MMM score, suggesting m^6^A modification status is a poor prognostic factor for predicting disease development and therapy resistance. However, their findings also exhibited that the primary PCa harbors a paucity of m^6^A modified mRNAs as compared to the BPH, implying hypo m^6^A modification of mRNAs contributes to PCa initiation In this context, a discrepancy is found between the expression pattern of m^6^A regulators and the m^6^A levels when the comparison was made between BPH and primary PCa (Lu et al., 2023). We hypothesize that the activities of m^6^A regulators are inhibited by some proteins so that a hypo m^6^A levels are observed in PCa.

**TABLE 2:** The clinical value of each individual m^6^A regulator in PCa.

Name	PCa/N	CRPC/PCa	Means	Prognosis	References
METTL3	High	High	MeRIP-qPCR RT-qPCR Western Bloting, IHC	Poor	Cai et al. (2019), Yuan et al. (2020), Chen et al. (2021a), Ma et al. (2020), Li et al. (2020), Haigh et al. (2022), Mao et al. (2022), Li et al. (2023a), Cotter et al. (2021), Li et al. (2023b), Lothion-Roy et al. (2023)
METTL14	High	High	IHC	Poor	Wang et al. (2022a), Li et al. (2023b), Lothion-Roy et al. (2023)
FTO	Low	NA	IHC,RT-qPCR Western Bloting	Good	Wang et al. (2022b), Zou et al. (2022), Li et al. (2022b), Azhati et al. (2023) Zhu et al. (2021)
ALKBH5	Low	NA	RT-qPCR, Western Bloting	Good	Li et al. (2023c)
YTHDF1	High	NA	IHC,Western Bloting,RT-qPCR	Poor	Li et al. (2021), Li et al. (2022b), Nie et al. (2023)
YTHDF2	High	NA	Western Bloting,RT-qPCR,IHC	Poor	Du et al. (2020) Li et al. (2020)
YTHDC1	NA	NA	NA	NA	NA
YTHDC2	High	NA	IHC,Western Bloting	Poor	Song et al. (2023), Ding et al. (2022)
IGF2BP1/2/3	NA	NA	NA	NA	NA
WTAP	High	NA	IHC,Western Bloting	NA	Lothion-Roy et al. (2023), Zhao et al. (2024)
HnRNP	High	NA	RT-qPCR, IHC	Poor	Wang et al. (2021), Cheng et al. (2023), Qi et al. (2023), Quan et al. (2023), Cheng et al. (2023)

According to this information, total m^6^A levels may serve as a diagnostic biomarker to predict disease status of PCa, and the elimination of m^6^A levels by METTL3/METTL14 inhibitor or others holds promise as a therapeutic strategy to prevent PCa progression. In 2021, Yankova et al identified a small molecule STM2457 as a potent METTL3 inhibitor to suppress acute myeloid leukaemia (AML), opening a new avenue of METTL3 targeted therapy. Additionally, Storm Therapeutics Company has screened another METTL3 inhibitor STC-15, which displays anti-tumor activity across different AML models and is currently being investigated in a clinical trail (NCT05584111) (Yankova et al., 2021). Although currently not available, it would be promising to test the efficacy of METTL3 inhibitors in PCa models and PCa patients. It is noting that some small molecules including curcumin (Chen et al., 2021b), quercetin (Zhu J. et al., 2023), epigallocatechin gallate (EGCG) (Wu et al., 2005) and simvastatin (Chen et al., 2020), have been reported to influence m^6^A signaling. However, in our opinion, they are not specific for interrupting m^6^A levels and their contributions to cancer prevention may not be solely due to the m^6^A alteration. Therefore, the continuous screening of METTL3-specific and potent inhibitors remains a priority for scientists and pharmacologists.

## 6 Conclusion

PCa is a male carcinoma and its mortality is continuously rising. Despite of the initial response, ADT treatment will lead to the emergence of recurrent tumor, suggesting other signaling pathways actively respond in order to bypass AR inhibition. As a type of epitranscriptomal modifications, m^6^A is now received much attention and it is indeed implicated into a variety of biological processes including tumorigenesis. Particularly in PCa, abnormal expression levels of m^6^A regulators are frequently observed by many researchers. The experimental evidence suggest that m^6^A writers, m^6^A erasers and m^6^A readers all contribute to PCa survival and malignancy. Additional evidence also suggest that the total m^6^A levels and METTL3 are closely related to enzalutamide resistance. These findings provide a strong rationale to propose a therapy using m^6^A inhibitor, alone or with anti-androgen, to treat CRPC patients.

Although numerous RNAs has been identified to be m^6^A modified, the blueprint of m^6^A signaling remains incomplete. In the clinical setting, a comprehensive understanding of m^6^A targets and their related signaling pathways can guide the discovery of novel targeted therapies to overcome PCa development. To this end, scientists should exert significant efforts to identify functional m^6^A targets during PCa evolution.

Although FTO inhibitors such as bisisantrene (Su et al., 2020), brequinar (Su et al., 2020), and Dac51 (Wu et al., 2023; Yang and Al-Hendy, 2023; Huang Y. et al., 2023; Liu Y. et al., 2021) have shown potency against several solid tumors, including renal carcinoma, bladder cancer, they may not be the ideal choice for the treatment of PCa model as researchers have confirmed the tumor suppressing role of FTO in PCa models. Alternatively, researchers should consider screening specific inhibitors of m^6^A readers, as they are positively implicated in PCa development. YTH family proteins, IGF2BP proteins, and other m^6^A readers have been proven to be oncogenic factors driving PCa progression. From our perspective, m^6^A reader inhibitors may be more specific than METTL3 inhibitors in suppressing a small population of RNA. While METTL3 has a variety of targets, each m^6^A reader has its uniquely recognized RNA population. Indeed, IGF2BP1 inhibitors (AVJ16 (Singh et al., 2024), BTYNB (Müller et al., 2020; Mahapatra et al., 2017; Jamal et al., 2023; Hagemann et al., 2023; Xiao et al., 2023; Wang JJ. et al., 2023), and 7773 (Singh et al., 2024)), IGF2BP2 inhibitor CWI1-2 (Weng et al., 2022), and YTHDF proteins inhibitor ebselen (Micaelli et al., 2022) all show promising anti-cancer activity in preclinical models. However, the identification of m^6^A reader inhibitors is still in the preliminary stage and requires intensive dedication.

## Figures and Tables

**Table:** 

ARv7	Androgen receptor variant 7
EGR-2	Early growth response protein 2
MeRIP	Methylated RNA Immunoprecipitation
ADT	Androgen deprivation therapy
FTO	Fat mass and obesity-associated
ALKBH5	Human Alk B homolog 5
c-MYC	c-Myelocytomatosis
USP4	Ubiquitin specific protease 4
LEF1	Lymphoid Enhancer Binding Factor 1
PRSS8	Serine protease 8
ZFHX3	Zinc-Finger Homeobox 3
NEAT1	Nuclear paraspeckle assembly transcript 1
MALAT1	Metastasis Associated Lung Adenocarcinoma Transcript 1
PVT1	Plasmacytoma variant translocation 1
DDIT4	DNA damage-inducible transcript 4
ABCC4	ATP binding cassette subfamily C member 4
RBM33	RNA Binding Motif Protein 33
YTHDF2	YTH N6-Methyladenosine RNA Binding Protein F2
ARHGDIA	Rho GDP Dissociation Inhibitor Alpha
SNHG7	Small nucleolar RNA hostgene 7
SRSF1	Serine And Arginine Rich Splicing Factor 1
MAPK	Mitogen activated protein kinase
MEK2	MAP kinase kinase 2
MALAT1	Metastasis associated in lung denocarcinoma transcript 1
ZNF217	Zinc Finger Protein 217
SIAH1	Siah E3 Ubiquitin Protein Ligase 1
TRIM44	Tripartite motif-containing 44
ELK1	ETS Transcription Factor 1
PLK1	Polo-like Kinase 1
NKX3-1	Neurokinin-3 Homeobox 1
KDM5A	Lysine-specific demethylase 5A
YTHDC1	YTH N6-Methyladenosine RNA Binding Protein C1
CD44v5	CD44 Vriant 5
SLC12A5	Solute Carrier Family 12 Member 5
HOXBI3	Homeobox B 13
IGF2BPs	Insulin-like growth factor 2 mRNA-binding proteins
CXCR4	Chemokine receptor type 4
CCAR1	Cell-cycle and apoptosis regulator 1
LDHA	Lactate Dehydrogenase A
ARHGAP29	Rho GTPase activating protein 29
PCAT6	Prostate cancer associated transcript 6
IGF1R	Insulin-like growth factor 1 receptor
VIM-AS1	Vimentin antisense RNA 1
HDAC4	Histone deacetylase 4
ERK	Extracellular regulated protein kinases
FAS	Fas cell surface death receptor
TP53INP1	Tumor Protein P53 Inducible Nuclear Protein 1
SESN2	Sestrin2
MDM2	Murine double minute2
RBM15	RNA Binding Motif Protein 15
AZGP1P2	Zinc-alpha 2-glycoprotein pseudogene 2
PDE5A	Phosphodiesterase 5A
MeRIP-seq	Methylated RNA Immunoprecipitation sequencing
